# 3-Cyano-11-oxo-3,4-seco-12a-aza-*C*-homoolean-4(23)-en-28-oic acid methyl ester

**DOI:** 10.1107/S1600536812002863

**Published:** 2012-01-31

**Authors:** A. Froelich, B. Bednarczyk-Cwynar, A. K. Gzella

**Affiliations:** a Department of Pharmaceutical Technology, Poznan University of Medical Sciences, ul. Grunwaldzka 6, 60-780 Poznań, Poland; b Department of Organic Chemistry, Poznan University of Medical Sciences, ul. Grunwaldzka 6, 60-780 Poznań, Poland; c Faculty of Pharmacy, Ludwik Rydygier Collegium Medicum in Bydgoszcz, Nicolaus Copernicus University in Torun, ul. M. Curie Skłodowskiej 9, 85-094 Bydgoszcz, Poland

## Abstract

The title compound, C_31_H_48_N_2_O_3_, is a Beckmann rearrangement product. The isopropenyl and meth­oxy­carbonyl groups have β-orientations, whereas the 2-cyano­ethyl group has an α-orientation. In the triterpenoid skeleton, the seven-membered lactam ring, as well as the three six-membered carbocyclic rings, have chair conformations. In the crystal, mol­ecules are linked *via* nonclassical C—H⋯O hydrogen bonds into layers parallel to the *ab* plane.

## Related literature

For ring-puckering parameters, see: Cremer & Pople (1975 ▶). For a related structure, see: Froelich & Gzella (2010 ▶). For bond-length data, see: Allen *et al.* (1987 ▶). For related literature on the Beckmann rearrangement reaction, see: Bednarczyk-Cwynar (2006 ▶).
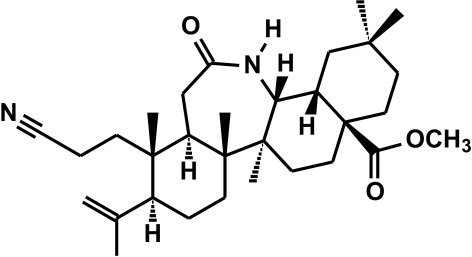


## Experimental

### 

#### Crystal data

C_31_H_48_N_2_O_3_
*M* *_r_* = 496.71
Monoclinic, 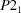

*a* = 6.8549 (10) Å
*b* = 11.711 (2) Å
*c* = 17.356 (3) Å
β = 91.607 (13)°
*V* = 1392.7 (4) Å^3^
*Z* = 2
Cu *K*α radiation
μ = 0.59 mm^−1^
*T* = 293 K
0.45 × 0.20 × 0.12 mm

#### Data collection

Kuma Diffraction KM-4 diffractometer
Absorption correction: ψ scan (North *et al.*, 1968 ▶) *T*_min_ = 0.830, *T*_max_ = 0.929
5219 measured reflections
5045 independent reflections
4815 reflections with *I* > 2σ(*I*)
*R*_int_ = 0.038
2 standard reflections every 100 reflections intensity decay: 2%

#### Refinement

*R*[*F* ^2^ > 2σ(*F* ^2^)] = 0.039
*wR*(*F* ^2^) = 0.112
*S* = 1.07
5045 reflections
337 parameters
1 restraint
H atoms treated by a mixture of independent and constrained refinement
Δρ_max_ = 0.19 e Å^−3^
Δρ_min_ = −0.18 e Å^−3^
Absolute structure: Flack (1983 ▶), 2248 Friedel pairs
Flack parameter: 0.0 (2)

### 

Data collection: *KM-4 Software* (Kuma Diffraction, 1996 ▶); cell refinement: *KM-4 Software*; data reduction: *KM-4 Software*; program(s) used to solve structure: *SHELXS97* (Sheldrick, 2008 ▶); program(s) used to refine structure: *SHELXL97* (Sheldrick, 2008 ▶); molecular graphics: *ORTEP-3 for Windows* (Farrugia, 1997 ▶); software used to prepare material for publication: *WinGX* (Farrugia, 1999 ▶), *PLATON* (Spek, 2009 ▶) and *enCIFer* (Allen *et al.*, 2004 ▶).

## Supplementary Material

Crystal structure: contains datablock(s) I, global. DOI: 10.1107/S1600536812002863/fj2500sup1.cif

Structure factors: contains datablock(s) I. DOI: 10.1107/S1600536812002863/fj2500Isup2.hkl

Additional supplementary materials:  crystallographic information; 3D view; checkCIF report

## Figures and Tables

**Table 1:** Hydrogen-bond geometry (Å, °)

*D*—H⋯*A*	*D*—H	H⋯*A*	*D*⋯*A*	*D*—H⋯*A*
C15—H15*B*⋯O2^i^	0.97	2.57	3.508 (3)	163
C31—H31*B*⋯O1^ii^	0.96	2.43	3.357 (3)	163

Symmetry codes: (i) 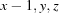; (ii) 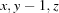.

**Fig. 1.**
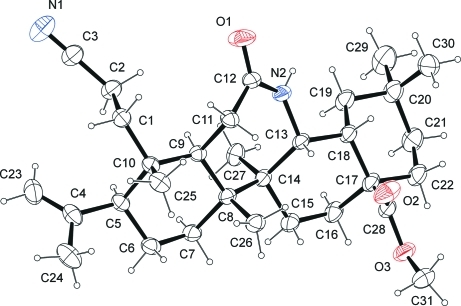
The molecular structure of (I) showing the atomic labelling scheme. Non-H atoms are drawn as 30% probability displacement ellipsoids; H atoms are shown as small spheres of arbitrary radius.

**Fig. 2.**
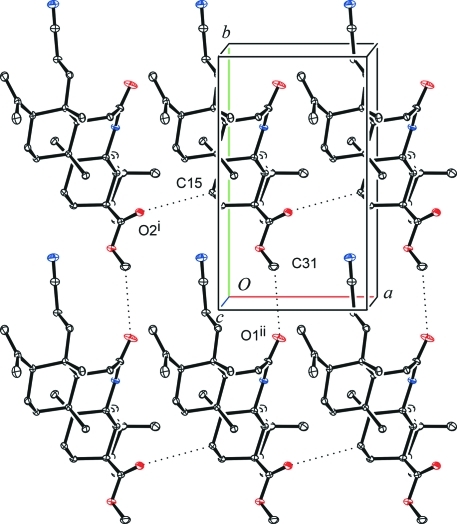
The hydrogen bonding (dotted lines) in the title structure. Symmetry codes: (i) -1 + x, y, z; (ii) x, -1 + y, z. The H atoms have been ommitted for clarity.

## supplementary crystallographic information

### Comment

The title compound was obtained from 3,12-dioxo-18*β*-olean-28-oic acid methyl ester as a product of the two-step synthesis. In the first step the diketone derivative mentioned above undergone the condensation with hydroxylamine hydrochloride to give the oxime derivative as a product. The latter reacted with POCl_3_ (Beckmann rearrangement reaction) (Bednarczyk-Cwynar, 2006). The results of the X-ray analysis showed that the final product is 3-cyano-11-oxo-3,4-seco-12*a*-aza-*C*-homoolean-4(23)-en-28-oic acid methyl ester, (I), (Fig. 1). Molecular structure obtained in the course of the X-ray investigation showed that oxime derivative formation and Beckmann rearrangement reaction took place within two triterpenoid rings, *i.e. A* and *C*. In ring *C* Beckmann rearrangement reaction took place while in ring *A* Beckmann fragmentation was observed.

As a result of Beckmann fragmentation C3—C4 bond cleavage and ring *A* opening were observed. In this process two new functions were formed. In C10 position 2-cyanoethyl group is observed. It reveals *α*-configuration and comprises of atoms C1, C2 and C3 of the original ring *A*. The linear fragment of this group consisting of atoms C2, C3 and N1 reveals conformation halfway between anticlinal and antiperiplanar (+*ac*/+*ap*) with respect to the C1—C10 bond [torsion angle C3—C2—C1—C10: 162.75 (17)°]. The C1—C2 bond is anticlinal (-*ac*) with respect to C5—C10 bond belonging to ring *B* [torsion angle C2—C1—C10—C25: 174.38 (15)°]. The other function formed as the result of the cleavage of ring *A* consists of atoms C4, C23 and C24. They form almost planar group (r.m.s. = 0.012 Å) along with C5 atom belonging to ring *B*. The dihedral angle between the mean plane of the new group and the least-squares plane of ring *B* is 67.10 (8)°. The C4═C23 double bond in isopropenyl residue reveals conformation halfway between synclinal and anticlinal (+*sc*/+*ac*) [torsion angle C23—C4—C5—C10: 93.3 (2)°]. The angular orientation of isopropenyl group described above is most probably caused by the sterical hindrance created by the cyanoethyl group.

The axial methyl group C25 adopts *β*-orientation while hydrogen atom in C5 position reveals *α*-orientation. Thus, both of these substituents retain the orientation observed in oleanolic acid molecules (Froelich & Gzella, 2010).

In the molecule of (I) the original six-membered carbocyclic ring *C* has been transformed into the seven-membered lactam ring in which nitrogen atom connects carbonyl group (C12═O1) and tertiary carbon atom C13.

The C12—N2 bond distance of 1.338 (2) Å is comparable with the normal length of the single (*C**—)NH—*C*(═*O*) bond in secondary amide which is 1.334 (1) Å (Allen *et al.*, 1987).

Seven-membered lactam ring adopts chair conformation {Cremer & Pople (1975) parameters: *Q*(2) = 0.388 (2) Å, *Q*(3) = 0.695 (2) Å, *φ*(2) = 319.8 (3)°, *φ*(3) = 282.53 (15)°}, as well as six-membered rings *B*, *D* and *E*.

Rings *B*/*C* and *C*/*D* are *trans*-fused [the dihedral angles 16.63 (10) and 19.07 (10)°, respectively], while rings *D*/*E* are *cis*-fused [the dihedral angle 56.69 (7)°]

The planar ester group in C17 is attached axially to ring *D* and equatorially to ring *E*. Its carbonyl (C28═O2) group is synperiplanar (-*sp*) with respect to C17—C18 bond belonging to both *D* and *E* rings [torsion angle C18—C17—C28—O2: -6.5 (3)°].

In the crystal lattice of (I) molecules are linked by nonclasical hydrogen bonds C15—H15B···O2^i^ and C31—H31B···O1^ii^ (Tab. 1, Fig. 2) into layers parallel to the *ab* plane.

In the molecule of (I) fourteen short H···H contacts are observed. The distances between related hydrogen atoms lie within the range of 1.92 - 2.20 Å. The short contacts are mainly the consequence of the presence of axial methyl groups C25, C26 and C27.

### Experimental

The title compound was synthesized according to the procedure described by Bednarczyk-Cwynar (2006) and dissolved in hot ethanol. The solution was set aside to crystallize at room temperature. After a week block-shaped colourless single crystals suitable for X-ray experiments were obtained.

### Refinement

Except for the amide H atom which was refined freely the remaining H atoms were placed in the idealized positions and were refined within the riding model approximation: C_methyl_—H = 0.96 Å, C_methylene_—H = 0.97 Å, C_methine_—H = 0.98 Å, C(*sp*^2^)—H = 0.93 Å; *U*_iso_(H) = 1.2*U*_eq_(C) or 1.5*U*_eq_(C) for methyl H. The methyl groups were refined as rigid groups which were allowed to rotate.

### Crystal data

**Table:**

C_31_H_48_N_2_O_3_	*F*(000) = 544
*M**_r_* = 496.71	*D*_x_ = 1.184 Mg m^−^^3^
Monoclinic, *P*2_1_	Melting point = 532–535 K
Hall symbol: P 2yb	Cu *K*α radiation, λ = 1.54178 Å
*a* = 6.8549 (10) Å	Cell parameters from 45 reflections
*b* = 11.711 (2) Å	θ = 15.5–28.6°
*c* = 17.356 (3) Å	µ = 0.59 mm^−^^1^
β = 91.607 (13)°	*T* = 293 K
*V* = 1392.7 (4) Å^3^	Block, colourless
*Z* = 2	0.45 × 0.20 × 0.12 mm

### Data collection

**Table:**

Kuma Diffraction KM-4 diffractometer	4815 reflections with *I* > 2σ(*I*)
Radiation source: fine-focus sealed tube, Kuma Diffraction	*R*_int_ = 0.038
graphite	θ_max_ = 70.2°, θ_min_ = 2.6°
ω–2θ scans	*h* = −8→8
Absorption correction: ψ scan (North *et al.*, 1968)	*k* = −14→14
*T*_min_ = 0.830, *T*_max_ = 0.929	*l* = 0→21
5219 measured reflections	2 standard reflections every 100 reflections
5045 independent reflections	intensity decay: 2%

### Refinement

**Table:**

Refinement on *F*^2^	Hydrogen site location: inferred from neighbouring sites
Least-squares matrix: full	H atoms treated by a mixture of independent and constrained refinement
*R*[*F*^2^ > 2σ(*F*^2^)] = 0.039	*w* = 1/[σ^2^(*F*_o_^2^) + (0.0684*P*)^2^ + 0.1756*P*] where *P* = (*F*_o_^2^ + 2*F*_c_^2^)/3
*wR*(*F*^2^) = 0.112	(Δ/σ)_max_ < 0.001
*S* = 1.07	Δρ_max_ = 0.19 e Å^−^^3^
5045 reflections	Δρ_min_ = −0.18 e Å^−^^3^
337 parameters	Extinction correction: *SHELXL97* (Sheldrick, 2008), Fc^*^=kFc[1+0.001xFc^2^λ^3^/sin(2θ)]^-1/4^
1 restraint	Extinction coefficient: 0.0184 (10)
Primary atom site location: structure-invariant direct methods	Absolute structure: Flack (1983), 2248 Friedel pairs
Secondary atom site location: difference Fourier map	Flack parameter: 0.0 (2)

### Special details

**Table:**

Geometry. All e.s.d.'s (except the e.s.d. in the dihedral angle between two l.s. planes) are estimated using the full covariance matrix. The cell e.s.d.'s are taken into account individually in the estimation of e.s.d.'s in distances, angles and torsion angles; correlations between e.s.d.'s in cell parameters are only used when they are defined by crystal symmetry. An approximate (isotropic) treatment of cell e.s.d.'s is used for estimating e.s.d.'s involving l.s. planes.
Refinement. Refinement of *F*^2^ against ALL reflections. The weighted *R*-factor *wR* and goodness of fit *S* are based on *F*^2^, conventional *R*-factors *R* are based on *F*, with *F* set to zero for negative *F*^2^. The threshold expression of *F*^2^ > σ(*F*^2^) is used only for calculating *R*-factors(gt) *etc*. and is not relevant to the choice of reflections for refinement. *R*-factors based on *F*^2^ are statistically about twice as large as those based on *F*, and *R*- factors based on ALL data will be even larger.

### Fractional atomic coordinates and isotropic or equivalent isotropic displacement parameters (Å^2^)

**Table:**

	*x*	*y*	*z*	*U*_iso_*/*U*_eq_	
O1	0.3886 (4)	0.87517 (15)	0.71870 (10)	0.0965 (7)	
O2	0.4487 (2)	0.37102 (14)	0.72260 (12)	0.0792 (5)	
O3	0.2567 (2)	0.22641 (11)	0.68910 (9)	0.0656 (4)	
N1	−0.1263 (4)	1.19826 (19)	0.83379 (15)	0.0912 (7)	
N2	0.2826 (2)	0.70494 (13)	0.67747 (10)	0.0504 (4)	
H2	0.314 (4)	0.726 (3)	0.6355 (18)	0.084 (9)*	
C1	−0.0113 (3)	0.92218 (16)	0.88714 (10)	0.0484 (4)	
H1A	−0.0693	0.9579	0.9314	0.058*	
H1B	0.1275	0.9384	0.8901	0.058*	
C2	−0.0969 (3)	0.97888 (16)	0.81417 (11)	0.0562 (5)	
H2A	−0.0133	0.9633	0.7712	0.067*	
H2B	−0.2245	0.9467	0.8021	0.067*	
C3	−0.1146 (4)	1.10237 (19)	0.82451 (13)	0.0641 (5)	
C4	−0.3546 (3)	0.8199 (2)	0.97148 (12)	0.0584 (5)	
C5	−0.2600 (2)	0.76406 (15)	0.90290 (10)	0.0449 (4)	
H5	−0.3268	0.7956	0.8570	0.054*	
C6	−0.2932 (3)	0.63565 (16)	0.89948 (11)	0.0500 (4)	
H6A	−0.2134	0.5986	0.9392	0.060*	
H6B	−0.4289	0.6191	0.9093	0.060*	
C7	−0.2416 (2)	0.58875 (16)	0.82117 (11)	0.0495 (4)	
H7A	−0.3225	0.6260	0.7819	0.059*	
H7B	−0.2716	0.5078	0.8197	0.059*	
C8	−0.0243 (2)	0.60564 (14)	0.80163 (10)	0.0420 (4)	
C9	0.0337 (2)	0.73273 (13)	0.81839 (9)	0.0388 (3)	
H9	−0.0265	0.7767	0.7760	0.047*	
C10	−0.0390 (2)	0.79123 (15)	0.89403 (9)	0.0418 (3)	
C11	0.2557 (2)	0.75245 (16)	0.81192 (10)	0.0466 (4)	
H11A	0.2953	0.8133	0.8469	0.056*	
H11B	0.3240	0.6836	0.8282	0.056*	
C12	0.3157 (3)	0.78280 (16)	0.73271 (11)	0.0521 (4)	
C13	0.2185 (2)	0.58645 (13)	0.68730 (10)	0.0419 (4)	
H13	0.2982	0.5558	0.7303	0.050*	
C14	0.0016 (2)	0.57437 (14)	0.71198 (10)	0.0429 (4)	
C15	−0.0613 (3)	0.44809 (16)	0.70120 (13)	0.0538 (4)	
H15A	−0.0046	0.4037	0.7434	0.065*	
H15B	−0.2020	0.4437	0.7049	0.065*	
C16	−0.0041 (3)	0.39319 (16)	0.62579 (13)	0.0578 (5)	
H16A	−0.0676	0.4334	0.5832	0.069*	
H16B	−0.0492	0.3147	0.6246	0.069*	
C17	0.2165 (3)	0.39543 (14)	0.61578 (12)	0.0501 (4)	
C18	0.2841 (3)	0.52108 (15)	0.61409 (11)	0.0469 (4)	
H18	0.4270	0.5187	0.6177	0.056*	
C19	0.2329 (3)	0.57702 (17)	0.53612 (11)	0.0590 (5)	
H19A	0.2837	0.6543	0.5364	0.071*	
H19B	0.0920	0.5819	0.5302	0.071*	
C20	0.3130 (4)	0.51322 (19)	0.46598 (12)	0.0624 (5)	
C21	0.2325 (4)	0.3926 (2)	0.46791 (14)	0.0715 (6)	
H21A	0.0920	0.3956	0.4597	0.086*	
H21B	0.2861	0.3494	0.4258	0.086*	
C22	0.2779 (3)	0.33091 (18)	0.54247 (14)	0.0646 (5)	
H22A	0.4173	0.3167	0.5462	0.077*	
H22B	0.2127	0.2574	0.5410	0.077*	
C23	−0.4447 (3)	0.9196 (2)	0.96374 (17)	0.0760 (7)	
H23A	−0.5091	0.9510	1.0052	0.091*	
H23B	−0.4436	0.9581	0.9169	0.091*	
C24	−0.3574 (4)	0.7587 (3)	1.04708 (13)	0.0835 (8)	
H24A	−0.4138	0.8073	1.0850	0.125*	
H24B	−0.2264	0.7392	1.0631	0.125*	
H24C	−0.4337	0.6903	1.0416	0.125*	
C25	0.0781 (2)	0.75743 (19)	0.96750 (10)	0.0543 (5)	
H25A	0.2152	0.7610	0.9578	0.081*	
H25B	0.0441	0.6811	0.9821	0.081*	
H25C	0.0481	0.8091	1.0084	0.081*	
C26	0.0951 (3)	0.52295 (17)	0.85416 (12)	0.0538 (4)	
H26A	0.1185	0.5578	0.9036	0.081*	
H26B	0.2176	0.5065	0.8310	0.081*	
H26C	0.0234	0.4533	0.8604	0.081*	
C27	−0.1286 (3)	0.64964 (18)	0.65913 (11)	0.0548 (4)	
H27A	−0.1193	0.6242	0.6068	0.082*	
H27B	−0.0862	0.7276	0.6630	0.082*	
H27C	−0.2616	0.6441	0.6746	0.082*	
C28	0.3206 (3)	0.33311 (16)	0.68200 (13)	0.0541 (4)	
C29	0.2405 (6)	0.5741 (3)	0.39255 (14)	0.0924 (9)	
H29A	0.2930	0.5369	0.3484	0.139*	
H29B	0.2826	0.6523	0.3939	0.139*	
H29C	0.1006	0.5712	0.3893	0.139*	
C30	0.5346 (4)	0.5125 (2)	0.46669 (15)	0.0751 (6)	
H30A	0.5832	0.4725	0.5115	0.113*	
H30B	0.5821	0.5896	0.4678	0.113*	
H30C	0.5787	0.4750	0.4212	0.113*	
C31	0.3545 (4)	0.1580 (2)	0.74689 (16)	0.0755 (6)	
H31A	0.4916	0.1751	0.7478	0.113*	
H31B	0.3353	0.0786	0.7351	0.113*	
H31C	0.3022	0.1746	0.7964	0.113*	

### Atomic displacement parameters (Å^2^)

**Table:**

	*U*^11^	*U*^22^	*U*^33^	*U*^12^	*U*^13^	*U*^23^
O1	0.163 (2)	0.0509 (9)	0.0781 (11)	−0.0462 (11)	0.0488 (12)	−0.0174 (8)
O2	0.0640 (8)	0.0526 (8)	0.1192 (14)	−0.0056 (7)	−0.0295 (9)	0.0127 (9)
O3	0.0747 (9)	0.0372 (7)	0.0850 (10)	−0.0067 (6)	0.0025 (7)	0.0040 (7)
N1	0.1190 (19)	0.0544 (12)	0.0995 (17)	0.0080 (12)	−0.0102 (13)	−0.0030 (12)
N2	0.0647 (9)	0.0359 (8)	0.0514 (9)	−0.0096 (7)	0.0137 (7)	−0.0032 (7)
C1	0.0469 (8)	0.0499 (10)	0.0484 (9)	−0.0059 (7)	0.0021 (7)	−0.0074 (8)
C2	0.0689 (11)	0.0442 (10)	0.0554 (10)	−0.0012 (8)	0.0012 (8)	0.0003 (8)
C3	0.0760 (13)	0.0505 (12)	0.0658 (12)	−0.0003 (10)	0.0005 (10)	−0.0003 (10)
C4	0.0417 (8)	0.0764 (14)	0.0574 (10)	−0.0092 (9)	0.0090 (7)	−0.0123 (10)
C5	0.0358 (7)	0.0533 (10)	0.0455 (8)	−0.0014 (7)	0.0021 (6)	0.0021 (8)
C6	0.0387 (8)	0.0535 (10)	0.0581 (10)	−0.0040 (7)	0.0052 (7)	0.0099 (8)
C7	0.0395 (8)	0.0430 (9)	0.0663 (11)	−0.0070 (7)	0.0043 (7)	0.0014 (8)
C8	0.0368 (7)	0.0372 (8)	0.0520 (9)	−0.0013 (6)	−0.0001 (6)	0.0034 (7)
C9	0.0356 (7)	0.0385 (8)	0.0421 (8)	−0.0017 (6)	−0.0005 (6)	0.0034 (6)
C10	0.0368 (7)	0.0466 (9)	0.0420 (8)	−0.0027 (6)	0.0016 (6)	0.0015 (7)
C11	0.0382 (7)	0.0499 (10)	0.0520 (9)	−0.0083 (7)	0.0039 (6)	−0.0052 (8)
C12	0.0593 (10)	0.0396 (9)	0.0583 (10)	−0.0107 (8)	0.0150 (8)	−0.0049 (8)
C13	0.0444 (8)	0.0297 (8)	0.0515 (9)	−0.0032 (6)	0.0013 (7)	−0.0017 (7)
C14	0.0386 (8)	0.0343 (8)	0.0555 (9)	0.0009 (6)	−0.0019 (6)	−0.0026 (7)
C15	0.0400 (8)	0.0403 (9)	0.0811 (12)	−0.0073 (7)	0.0012 (8)	−0.0086 (9)
C16	0.0484 (9)	0.0410 (10)	0.0836 (13)	−0.0042 (7)	−0.0053 (9)	−0.0159 (9)
C17	0.0519 (9)	0.0326 (8)	0.0658 (11)	−0.0014 (7)	0.0005 (8)	−0.0095 (8)
C18	0.0503 (9)	0.0349 (8)	0.0554 (10)	−0.0004 (7)	0.0022 (7)	−0.0054 (7)
C19	0.0803 (13)	0.0431 (10)	0.0535 (10)	0.0060 (9)	0.0029 (9)	−0.0057 (8)
C20	0.0824 (13)	0.0531 (11)	0.0517 (10)	0.0031 (10)	0.0019 (9)	−0.0118 (9)
C21	0.0860 (15)	0.0590 (14)	0.0694 (13)	−0.0053 (11)	0.0015 (11)	−0.0255 (11)
C22	0.0721 (12)	0.0412 (10)	0.0806 (14)	−0.0029 (9)	0.0046 (10)	−0.0187 (10)
C23	0.0614 (12)	0.0778 (16)	0.0898 (16)	0.0003 (11)	0.0181 (11)	−0.0285 (13)
C24	0.0759 (14)	0.120 (2)	0.0555 (12)	−0.0068 (15)	0.0195 (10)	−0.0030 (14)
C25	0.0429 (8)	0.0731 (13)	0.0466 (9)	−0.0053 (8)	−0.0033 (7)	0.0029 (9)
C26	0.0505 (9)	0.0480 (10)	0.0627 (11)	0.0046 (8)	0.0000 (8)	0.0135 (9)
C27	0.0556 (10)	0.0539 (11)	0.0542 (10)	0.0131 (8)	−0.0081 (8)	−0.0049 (8)
C28	0.0470 (9)	0.0358 (9)	0.0798 (13)	0.0019 (7)	0.0067 (8)	−0.0019 (8)
C29	0.134 (3)	0.086 (2)	0.0564 (13)	0.0207 (17)	−0.0029 (14)	−0.0086 (13)
C30	0.0873 (15)	0.0672 (14)	0.0718 (14)	−0.0075 (12)	0.0212 (11)	−0.0135 (11)
C31	0.0926 (16)	0.0482 (11)	0.0861 (15)	0.0054 (11)	0.0117 (13)	0.0144 (11)

### Geometric parameters (Å, °)

**Table:**

O1—C12	1.219 (2)	C15—H15B	0.9700
O2—C28	1.196 (3)	C16—C17	1.527 (3)
O3—C28	1.331 (2)	C16—H16A	0.9700
O3—C31	1.435 (3)	C16—H16B	0.9700
N1—C3	1.138 (3)	C17—C28	1.522 (3)
N2—C12	1.338 (2)	C17—C18	1.543 (2)
N2—C13	1.467 (2)	C17—C22	1.548 (3)
N2—H2	0.80 (3)	C18—C19	1.535 (3)
C1—C2	1.532 (3)	C18—H18	0.9800
C1—C10	1.550 (3)	C19—C20	1.542 (3)
C1—H1A	0.9700	C19—H19A	0.9700
C1—H1B	0.9700	C19—H19B	0.9700
C2—C3	1.463 (3)	C20—C21	1.517 (3)
C2—H2A	0.9700	C20—C30	1.519 (3)
C2—H2B	0.9700	C20—C29	1.531 (4)
C4—C23	1.326 (4)	C21—C22	1.507 (4)
C4—C24	1.495 (3)	C21—H21A	0.9700
C4—C5	1.519 (2)	C21—H21B	0.9700
C5—C6	1.522 (3)	C22—H22A	0.9700
C5—C10	1.560 (2)	C22—H22B	0.9700
C5—H5	0.9800	C23—H23A	0.9300
C6—C7	1.517 (3)	C23—H23B	0.9300
C6—H6A	0.9700	C24—H24A	0.9600
C6—H6B	0.9700	C24—H24B	0.9600
C7—C8	1.549 (2)	C24—H24C	0.9600
C7—H7A	0.9700	C25—H25A	0.9600
C7—H7B	0.9700	C25—H25B	0.9600
C8—C26	1.548 (2)	C25—H25C	0.9600
C8—C9	1.566 (2)	C26—H26A	0.9600
C8—C14	1.613 (2)	C26—H26B	0.9600
C9—C11	1.547 (2)	C26—H26C	0.9600
C9—C10	1.574 (2)	C27—H27A	0.9600
C9—H9	0.9800	C27—H27B	0.9600
C10—C25	1.540 (2)	C27—H27C	0.9600
C11—C12	1.489 (2)	C29—H29A	0.9600
C11—H11A	0.9700	C29—H29B	0.9600
C11—H11B	0.9700	C29—H29C	0.9600
C13—C18	1.560 (2)	C30—H30A	0.9600
C13—C14	1.565 (2)	C30—H30B	0.9600
C13—H13	0.9800	C30—H30C	0.9600
C14—C27	1.539 (2)	C31—H31A	0.9600
C14—C15	1.550 (2)	C31—H31B	0.9600
C15—C16	1.519 (3)	C31—H31C	0.9600
C15—H15A	0.9700		
			
C28—O3—C31	116.11 (18)	C17—C16—H16B	109.3
C12—N2—C13	127.35 (16)	H16A—C16—H16B	107.9
C12—N2—H2	114 (2)	C28—C17—C16	110.53 (17)
C13—N2—H2	119 (2)	C28—C17—C18	109.67 (15)
C2—C1—C10	116.57 (14)	C16—C17—C18	108.51 (14)
C2—C1—H1A	108.1	C28—C17—C22	104.70 (15)
C10—C1—H1A	108.1	C16—C17—C22	112.22 (16)
C2—C1—H1B	108.1	C18—C17—C22	111.15 (16)
C10—C1—H1B	108.1	C19—C18—C17	111.23 (15)
H1A—C1—H1B	107.3	C19—C18—C13	116.38 (14)
C3—C2—C1	110.99 (17)	C17—C18—C13	111.02 (14)
C3—C2—H2A	109.4	C19—C18—H18	105.8
C1—C2—H2A	109.4	C17—C18—H18	105.8
C3—C2—H2B	109.4	C13—C18—H18	105.8
C1—C2—H2B	109.4	C18—C19—C20	114.29 (16)
H2A—C2—H2B	108.0	C18—C19—H19A	108.7
N1—C3—C2	178.7 (3)	C20—C19—H19A	108.7
C23—C4—C24	119.6 (2)	C18—C19—H19B	108.7
C23—C4—C5	120.5 (2)	C20—C19—H19B	108.7
C24—C4—C5	119.8 (2)	H19A—C19—H19B	107.6
C4—C5—C6	112.90 (15)	C21—C20—C30	111.0 (2)
C4—C5—C10	115.24 (14)	C21—C20—C29	110.0 (2)
C6—C5—C10	110.01 (14)	C30—C20—C29	108.1 (2)
C4—C5—H5	106.0	C21—C20—C19	107.21 (19)
C6—C5—H5	106.0	C30—C20—C19	111.98 (19)
C10—C5—H5	106.0	C29—C20—C19	108.49 (19)
C7—C6—C5	110.75 (15)	C22—C21—C20	113.50 (18)
C7—C6—H6A	109.5	C22—C21—H21A	108.9
C5—C6—H6A	109.5	C20—C21—H21A	108.9
C7—C6—H6B	109.5	C22—C21—H21B	108.9
C5—C6—H6B	109.5	C20—C21—H21B	108.9
H6A—C6—H6B	108.1	H21A—C21—H21B	107.7
C6—C7—C8	113.56 (14)	C21—C22—C17	114.66 (17)
C6—C7—H7A	108.9	C21—C22—H22A	108.6
C8—C7—H7A	108.9	C17—C22—H22A	108.6
C6—C7—H7B	108.9	C21—C22—H22B	108.6
C8—C7—H7B	108.9	C17—C22—H22B	108.6
H7A—C7—H7B	107.7	H22A—C22—H22B	107.6
C26—C8—C7	106.68 (14)	C4—C23—H23A	120.0
C26—C8—C9	110.97 (14)	C4—C23—H23B	120.0
C7—C8—C9	108.74 (13)	H23A—C23—H23B	120.0
C26—C8—C14	110.82 (14)	C4—C24—H24A	109.5
C7—C8—C14	108.33 (13)	C4—C24—H24B	109.5
C9—C8—C14	111.15 (13)	H24A—C24—H24B	109.5
C11—C9—C8	111.94 (13)	C4—C24—H24C	109.5
C11—C9—C10	109.32 (12)	H24A—C24—H24C	109.5
C8—C9—C10	118.94 (13)	H24B—C24—H24C	109.5
C11—C9—H9	105.1	C10—C25—H25A	109.5
C8—C9—H9	105.1	C10—C25—H25B	109.5
C10—C9—H9	105.1	H25A—C25—H25B	109.5
C25—C10—C1	104.82 (14)	C10—C25—H25C	109.5
C25—C10—C5	110.57 (14)	H25A—C25—H25C	109.5
C1—C10—C5	109.34 (14)	H25B—C25—H25C	109.5
C25—C10—C9	114.13 (14)	C8—C26—H26A	109.5
C1—C10—C9	108.89 (13)	C8—C26—H26B	109.5
C5—C10—C9	108.95 (12)	H26A—C26—H26B	109.5
C12—C11—C9	113.62 (15)	C8—C26—H26C	109.5
C12—C11—H11A	108.8	H26A—C26—H26C	109.5
C9—C11—H11A	108.8	H26B—C26—H26C	109.5
C12—C11—H11B	108.8	C14—C27—H27A	109.5
C9—C11—H11B	108.8	C14—C27—H27B	109.5
H11A—C11—H11B	107.7	H27A—C27—H27B	109.5
O1—C12—N2	121.62 (18)	C14—C27—H27C	109.5
O1—C12—C11	121.43 (17)	H27A—C27—H27C	109.5
N2—C12—C11	116.94 (16)	H27B—C27—H27C	109.5
N2—C13—C18	105.97 (13)	O2—C28—O3	122.1 (2)
N2—C13—C14	114.04 (13)	O2—C28—C17	126.02 (18)
C18—C13—C14	118.44 (13)	O3—C28—C17	111.80 (17)
N2—C13—H13	105.8	C20—C29—H29A	109.5
C18—C13—H13	105.8	C20—C29—H29B	109.5
C14—C13—H13	105.8	H29A—C29—H29B	109.5
C27—C14—C15	108.65 (15)	C20—C29—H29C	109.5
C27—C14—C13	108.93 (15)	H29A—C29—H29C	109.5
C15—C14—C13	108.43 (13)	H29B—C29—H29C	109.5
C27—C14—C8	111.55 (13)	C20—C30—H30A	109.5
C15—C14—C8	107.18 (14)	C20—C30—H30B	109.5
C13—C14—C8	111.98 (12)	H30A—C30—H30B	109.5
C16—C15—C14	115.47 (16)	C20—C30—H30C	109.5
C16—C15—H15A	108.4	H30A—C30—H30C	109.5
C14—C15—H15A	108.4	H30B—C30—H30C	109.5
C16—C15—H15B	108.4	O3—C31—H31A	109.5
C14—C15—H15B	108.4	O3—C31—H31B	109.5
H15A—C15—H15B	107.5	H31A—C31—H31B	109.5
C15—C16—C17	111.78 (15)	O3—C31—H31C	109.5
C15—C16—H16A	109.3	H31A—C31—H31C	109.5
C17—C16—H16A	109.3	H31B—C31—H31C	109.5
C15—C16—H16B	109.3		
			
C10—C1—C2—C3	162.75 (17)	C26—C8—C14—C27	175.55 (15)
C23—C4—C5—C6	−139.1 (2)	C7—C8—C14—C27	58.84 (18)
C24—C4—C5—C6	37.0 (2)	C9—C8—C14—C27	−60.56 (17)
C23—C4—C5—C10	93.3 (2)	C26—C8—C14—C15	56.73 (17)
C24—C4—C5—C10	−90.6 (2)	C7—C8—C14—C15	−59.98 (16)
C4—C5—C6—C7	165.88 (14)	C9—C8—C14—C15	−179.38 (13)
C10—C5—C6—C7	−63.84 (18)	C26—C8—C14—C13	−62.06 (17)
C5—C6—C7—C8	62.0 (2)	C7—C8—C14—C13	−178.77 (14)
C6—C7—C8—C26	71.19 (19)	C9—C8—C14—C13	61.83 (16)
C6—C7—C8—C9	−48.55 (19)	C27—C14—C15—C16	73.06 (19)
C6—C7—C8—C14	−169.47 (14)	C13—C14—C15—C16	−45.2 (2)
C26—C8—C9—C11	54.52 (18)	C8—C14—C15—C16	−166.26 (14)
C7—C8—C9—C11	171.55 (14)	C14—C15—C16—C17	58.7 (2)
C14—C8—C9—C11	−69.29 (16)	C15—C16—C17—C28	58.8 (2)
C26—C8—C9—C10	−74.57 (17)	C15—C16—C17—C18	−61.5 (2)
C7—C8—C9—C10	42.47 (19)	C15—C16—C17—C22	175.26 (17)
C14—C8—C9—C10	161.63 (13)	C28—C17—C18—C19	162.91 (16)
C2—C1—C10—C25	174.38 (15)	C16—C17—C18—C19	−76.3 (2)
C2—C1—C10—C5	−67.07 (19)	C22—C17—C18—C19	47.6 (2)
C2—C1—C10—C9	51.9 (2)	C28—C17—C18—C13	−65.79 (18)
C4—C5—C10—C25	56.8 (2)	C16—C17—C18—C13	55.0 (2)
C6—C5—C10—C25	−72.26 (18)	C22—C17—C18—C13	178.91 (15)
C4—C5—C10—C1	−58.1 (2)	N2—C13—C18—C19	−48.9 (2)
C6—C5—C10—C1	172.83 (14)	C14—C13—C18—C19	80.7 (2)
C4—C5—C10—C9	−177.05 (15)	N2—C13—C18—C17	−177.45 (14)
C6—C5—C10—C9	53.93 (17)	C14—C13—C18—C17	−47.9 (2)
C11—C9—C10—C25	−52.17 (18)	C17—C18—C19—C20	−55.4 (2)
C8—C9—C10—C25	78.11 (18)	C13—C18—C19—C20	176.09 (16)
C11—C9—C10—C1	64.55 (17)	C18—C19—C20—C21	57.8 (2)
C8—C9—C10—C1	−165.18 (13)	C18—C19—C20—C30	−64.2 (3)
C11—C9—C10—C5	−176.28 (13)	C18—C19—C20—C29	176.6 (2)
C8—C9—C10—C5	−46.00 (18)	C30—C20—C21—C22	66.9 (3)
C8—C9—C11—C12	88.72 (17)	C29—C20—C21—C22	−173.5 (2)
C10—C9—C11—C12	−137.32 (15)	C19—C20—C21—C22	−55.7 (3)
C13—N2—C12—O1	172.1 (2)	C20—C21—C22—C17	53.6 (3)
C13—N2—C12—C11	−9.2 (3)	C28—C17—C22—C21	−166.17 (18)
C9—C11—C12—O1	116.4 (2)	C16—C17—C22—C21	73.9 (2)
C9—C11—C12—N2	−62.4 (2)	C18—C17—C22—C21	−47.8 (2)
C12—N2—C13—C18	−157.08 (18)	C31—O3—C28—O2	−1.8 (3)
C12—N2—C13—C14	70.8 (2)	C31—O3—C28—C17	176.20 (18)
N2—C13—C14—C27	48.48 (19)	C16—C17—C28—O2	−126.1 (2)
C18—C13—C14—C27	−77.27 (18)	C18—C17—C28—O2	−6.5 (3)
N2—C13—C14—C15	166.56 (15)	C22—C17—C28—O2	112.9 (2)
C18—C13—C14—C15	40.8 (2)	C16—C17—C28—O3	56.0 (2)
N2—C13—C14—C8	−75.39 (17)	C18—C17—C28—O3	175.59 (15)
C18—C13—C14—C8	158.86 (13)	C22—C17—C28—O3	−65.1 (2)

### Hydrogen-bond geometry (Å, °)

**Table:**

*D*—H···*A*	*D*—H	H···*A*	*D*···*A*	*D*—H···*A*
C13—H13···O2	0.98	2.40	3.029 (2)	121
C15—H15B···O2^i^	0.97	2.57	3.508 (3)	163
C31—H31B···O1^ii^	0.96	2.43	3.357 (3)	163

Symmetry codes: (i) *x*−1, *y*, *z*; (ii) *x*, *y*−1, *z*.

## References

[bb1] Allen, F. H., Johnson, O., Shields, G. P., Smith, B. R. & Towler, M. (2004). *J. Appl. Cryst.* **37**, 335–338.

[bb2] Allen, F. H., Kennard, O., Watson, D. G., Brammer, L., Orpen, A. G. & Taylor, R. (1987). *J. Chem. Soc. Perkin Trans. 2*, pp. S1–19.

[bb3] Bednarczyk-Cwynar, B. (2006). PhD thesis, Poznan University of Medical Sciences, Poznań, Poland.

[bb4] Cremer, D. & Pople, J. A. (1975). *J. Am. Chem. Soc.* **97**, 1354–1358.

[bb5] Farrugia, L. J. (1997). *J. Appl. Cryst.* **30**, 565.

[bb6] Farrugia, L. J. (1999). *J. Appl. Cryst.* **32**, 837–838.

[bb7] Flack, H. D. (1983). *Acta Cryst.* A**39**, 876–881.

[bb8] Froelich, A. & Gzella, A. K. (2010). *Acta Cryst.* E**66**, o2790. 10.1107/S1600536810039929 PMC3009164 21588987

[bb9] Kuma Diffraction (1996). *KM-4 Software* Kuma Diffraction, Wrocław, Poland.

[bb10] North, A. C. T., Phillips, D. C. & Mathews, F. S. (1968). *Acta Cryst.* A**24**, 351–359.

[bb11] Sheldrick, G. M. (2008). *Acta Cryst.* A**64**, 112–122. 10.1107/S0108767307043930 18156677

[bb12] Spek, A. L. (2009). *Acta Cryst.* D**65**, 148–155. 10.1107/S090744490804362X PMC2631630 19171970

